# Olfactory experiences dynamically regulate plasticity of dendritic spines in granule cells of *Xenopus* tadpoles *in vivo*

**DOI:** 10.1038/srep35009

**Published:** 2016-10-07

**Authors:** Li Zhang, Yubin Huang, Bing Hu

**Affiliations:** 1 Chinese Academy of Sciences Key Laboratory of Brain Function and Disease, and School of Life Sciences, University of Science and Technology of China, Hefei, Anhui Province, P. R. China

## Abstract

Granule cells, rich in dendrites with densely punctated dendritic spines, are the most abundant inhibitory interneurons in the olfactory bulb. The dendritic spines of granule cells undergo remodeling during the development of the nervous system. The morphological plasticity of the spines’ response to different olfactory experiences *in vivo* is not fully known. In initial studies, a single granule cell in *Xenopus* tadpoles was labeled with GFP plasmids via cell electroporation; then, morphologic changes of the granule cell spines were visualized by *in vivo* confocal time-lapse imaging. With the help of long-term imaging, the total spine density, dynamics, and stability of four types of dendritic spines (mushroom, stubby, thin and filopodia) were obtained. Morphological analysis demonstrated that odor enrichment produced a remarkable increase in the spine density and stability of large mushroom spine. Then, with the help of short-term imaging, we analyzed the morphological transitions among different spines. We found that transitions between small spines (thin and filopodia) were more easily influenced by odor stimulation or olfactory deprivation. These results indicate that different olfactory experiences can regulate the morphological plasticity of different dendritic spines in the granule cell.

Inhibitory interneurons in the central nervous system undergo structural remodeling during development^1^,^2^ and under sensory experience^3^,^4^, playing an important role in the regulation of neural circuits. Granule cells are the main interneurons in the olfactory bulb. They are rich in dendrites with densely punctated dendritic spines, and they release neurotransmitter γ-aminobutyric acid (GABA) to inhibit the principal excitatory afferent neurons, the mitral and tufted cells^5^,^6^.

Dendritic spines are the most common postsynaptic sites of the vast majority of excitatory synapses in the cortex, connecting presynaptic and postsynaptic neurons^7^. In most CNS neurons, the spines undergo morphological and physiological changes, during early development and in adulthood, correlating with their maturation and alterations in neuronal activity. The stability of spines determines the maturity of neural circuits and the nervous system, and the maturation of spines is related to learning and memory^8^,^9^. It has been reported that the dendritic spines of granule cells in the olfactory bulb are highly dynamic during development^2^,^10^, and this synaptic connectivity is vital to odor information processing and encoding.

Sensory experiences play an important role in regulating the synaptic plasticity of dendritic spines in the cortex. Long-term imaging experiments on rodents have provided support for the experience-dependent plasticity of spine growth and retraction in the barrel cortex, the visual cortex, and the motor cortex^11^,^12^,^13^. Although the olfactory bulb in vertebrates is conservative—divided into the classical six layers^14^—the morphology of internal interneurons is difficult to observe *in vivo* in rodents. In the *Xenopus laevis* tadpole, however, aside from the retinal ganglion cell (RGC) axon arbors^15^, the granule cells of the olfactory bulb can also be used as an *in vivo* imaging model for the observation of synapse dynamics due to the cephalo-caudal layer pattern of the olfactory bulb in early-stage development. Based on their morphology in slices, granule cells are the only reported spiny neurons in the olfactory bulb of *Xenopus*^16^,^17^. Therefore, it remains an open question whether, and how, olfactory experiences regulate the synaptic plasticity of granule cells in the olfactory bulb of *Xenopus in vivo*.

*In vivo* studies have reported that dendritic spines are highly motile^18^,^19^. Most dendritic spines perform rapid extension or retraction during synaptic structure development, and long-term imaging at day-long intervals might miss some of the details. Our previous study found that there were some preferences for the transformation of spines’ morphology from one category to another on timescales from minutes to hours^20^. However, little is known about the roles of odor stimulation and olfactory deprivation in determining these preferences.

Through single-cell electroporation, green fluorescent protein (GFP) plasmids are expressed in granule cells, allowing the structures of all types of spines to be observed *in vivo*. In this study, 1) we use long-term imaging to investigate whether odor enrichment has a regulative effect on synaptic structure plasticity, including the spine density, dynamics, and stability; 2) we use short-term imaging to investigate whether odor stimulation and olfactory deprivation play different roles in spines’ morphological transitioning.

## Results

### Odor environment changed stability and dynamics of dendritic spines in granule cells

**Figure 1:**
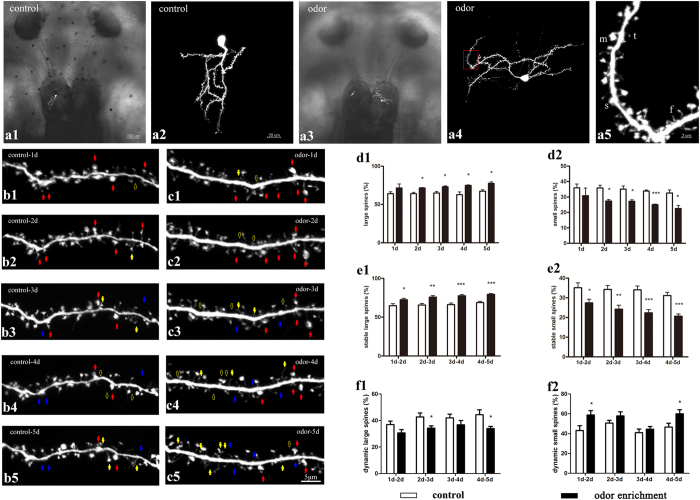
Stability and dynamics of dendritic spines during 5-day time-lapse imaging. (**a**) Each single granule cell in the odor and control groups was labeled by electroporation with GFP. Overlay images of bright field and fluorescence are shown in **a1** and **a3**, with amplification of fluorescence images in **a2** and **a4**. (**a5**) High magnification views of the four types of dendritic spines in a single granule cell are shown in **a4**. f indicates filopodia, t indicates thin spine, s indicates stubby spine, m indicates mushroom spine. (**b**,**c**) Serial time-lapse images of a single neuron each in the control and the odor groups at 1-day interval are shown. The red arrow indicates stable spines, yellow arrow indicates newly added spines, and blue arrow indicates eliminated spines. Scale bar corresponds to 5 μm. (**d**) Percentages of the large and small dendritic spines at 1-day interval in odor stimulation compared with the control group were analyzed. (**e**) The stability of large and small spines at 1-day intervals was analyzed. (**f**) The dynamic changes (added or eliminated) of large and small spines at 1-day intervals were analyzed. Error bars indicate the mean ± SEM. N = 6 neurons in 6 tadpoles, n = 18 dendrites in each group. Numbers of spines in control group are 952 at 1d, 962 at 2d, 973 at 3d, 1013 at 4d, and 1035 at 5d. Numbers of spines in odor group are 1164 at 1d, 1223 at 2d, 1357 at 3d, 1390 at 4d, and 1418 at 5d. The significance levels were *p < 0.05, **p < 0.01, and ***p < 0.001. Two-sample unpaired t-tests.

First, we wanted to know whether odor enrichment influenced the synaptic structure plasticity of the granule cell, including the dynamics and stability of large spines and small spines, respectively. Only tadpoles with a single labeled granule cell were stimulated by amino acid mixtures for 5 days. The dendritic spines of granule cells in the odor and control group were *in vivo* imaged at 1-day intervals (Fig. 1b,c serial). The stable and dynamic proportions of each type of spine were calculated and analyzed.

In proportion, large spines increased (p = 0.0165 at 2d, p = 0.0256 at 3d, p = 0.0313 at 4d, p = 0.0214 at 5d; Fig. 1d1) and small spines decreased (p = 0.0130 at 2d, p = 0.0254 at 3d, p = 0.0004 at 4d, p = 0.0222 at 5d; Fig. 1d2) after odor stimulation. The stability of the large spines significantly improved, while the stability of the small spines decreased (p = 0.0228 at 1d-2d, p = 0.0016 at 2d-3d, p = 0.0002 at 3d-4d, p < 0.0001 at 4d-5d; Fig. 1e). The percentage of the dynamic large spines tended to be lower (p = 0.0261 at 2d-3d, p = 0.0335 at 4d-5d; Fig. 1f1), and the percentage of the dynamic small spines tended to be higher (p = 0.0247 at 1d-2d, p = 0.0267 at 4d-5d; Fig. 1f2). These results suggest that the proportion of large spines increased because of an increase in large spine stabilization and the proportion of small spines decreased because of a decrease in small spine stabilization.

### The long-term structural plasticity of dendritic spines was positively and negatively regulated by odor-enriched and odor-removed environment

**Figure 2:**
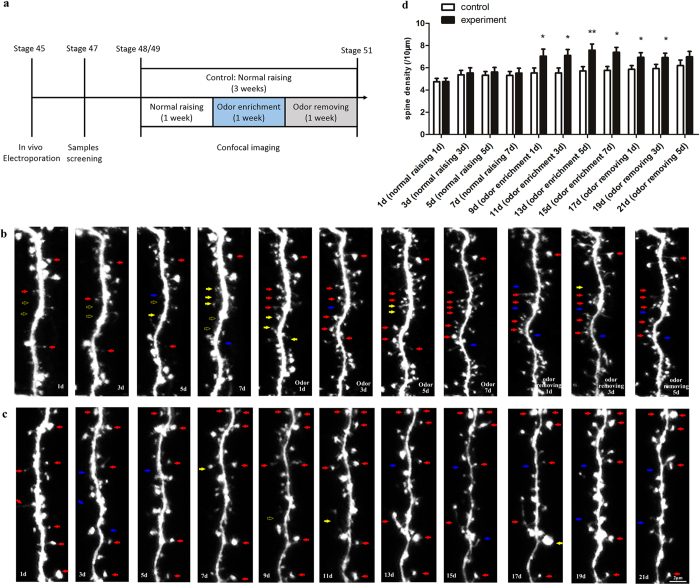
Long-term imaging of dendritic spines *in vivo*. (**a**) Olfactory manipulation process. (**b**,**c**) Time-lapse imaging of spines in experimental group (**b**, normally raising for 1 week, odor treating for 1 week, and odor removing for 1 week), compared with imaging of spines in control group (**c**, normal raising for 3 weeks’ development), showed sustained number changes in dendritic spines under the two conditions. The red arrow indicates the stable spines, yellow arrow indicates the newly added spines, and blue arrow indicates the eliminated spines. Scale bar corresponds to 2 μm. (**d**) Quantitative analysis of spine densities showed changed numbers of spines per 10 μm dendrite in the control and experimental groups. N = 6 neurons in 6 tadpoles and n = 21 dendrites were used for the 2-day interval observation. Numbers of spines in control group are 994 at 1d, 1123 at 3d, 1135 at 5d, 1182 at 7d, 1207 at 9d, 1214 at 11d, 1245 at 13d, 1271 at 15d, 1292 at 17d, 1267 at 19d, and 1360 at 21d. Numbers of spines in experimental group are 971 at 1d, 1164 at 3d, 1160 at 5d, 1169 at 7d, and 1525 at 9d, 1583 at 11d, 1654 at 13d, 1678 at 15d, 1599 at 17d, 1633 at 19d, and 1700 at 21d. Error bars indicate the mean ± SEM. The significance levels were *p < 0.05, **p < 0.01, and ***p < 0.001. Two-sample unpaired t-tests.

Next, in order to obtain detailed information on the long-term morphologic plasticity of the 4 types of spines in the same granule cell before odor, during odor, and after odor wash-out treatment, we treated the same tadpole in normal raising conditions for 1 week, then in odor enrichment for 1 week, and finally in odor-removing conditions for 1 week. In parallel, we used a time-point matched control group to show the natural development of dendritic spines over 3 weeks in normal raising conditions (Fig. 2a). Finally, we obtained the *in vivo* time-lapse images of spines in the control and experimental groups at 2-day intervals over 3 weeks (Fig. 2b,c).

Our *in vivo* confocal imaging showed that the development of neurons was accompanied by a gradual increase in the number of spines. The spine density of the control group increased gradually throughout the imaging process (Fig. 2d). During the first week, the spine density of the experimental group was similar to that of the control group. In the second week, after the initial odor bathing, the spine density in the experimental group was a little higher than in the control group (p = 0.0498 on the first day, p = 0.0301 on the third day, p = 0.0064 on the fifth day, p = 0.0165 on the seventh day). Even after odor removal for 3 days, the spine density remained a little higher than the control (p = 0.0491 on the first day, p = 0.0398 on the third day). These results show that the enriched environment induced an increase in the number of spines. However, there was no obvious change in the average length of dendrites (Supplementary Fig. S1b) or branching number and pattern (Supplementary Fig. S1a), indicating that the dendritic morphology of an individual granule cell was relatively stable throughout the course of the experiment.

**Figure 3:**
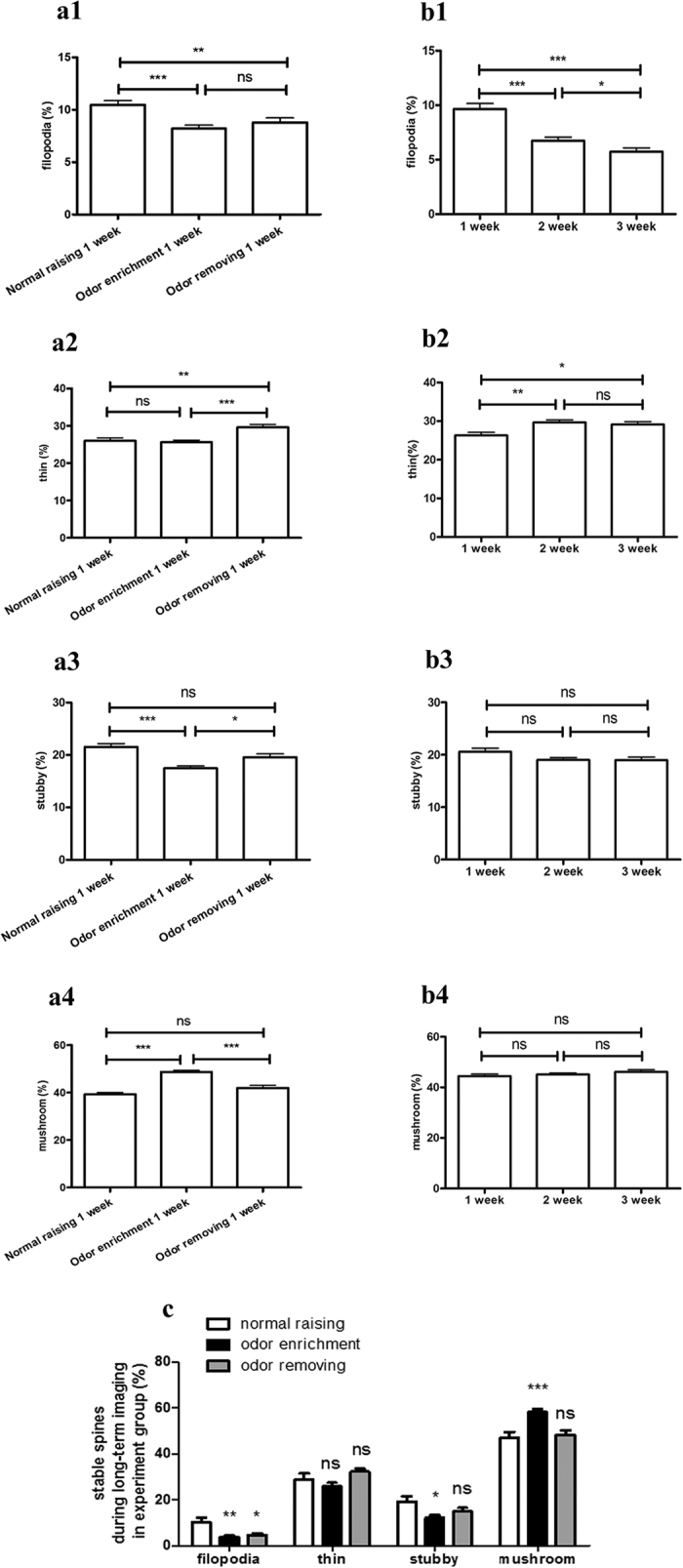
Ratio changes of the four spines influenced by odor-enriched and odor-removed environment. (**a**) Statistical analysis of filopodia (**a1**), thin (**a2**), stubby (**a3**), and mushroom (**a4**) spines in the experimental group showed the dynamic changing percentages of spines influenced by normal raising for 1 week, odor enrichment for 1 week, and odor removing for 1 week. (**b**) Respective percentages of the four types of spines during natural development for 3 weeks in the control group were analyzed, showing differences from the experimental group. (**c**) In the experimental group, the stability of each type of spine, respectively treated with normal raising, odor stimulation, and odor removal, was analyzed. N = 6 neurons in 6 tadpoles, n = 21 dendrites. Total numbers of spines in control group are 1109 at the 1^st^ week, 1234 at the 2^nd^ week, and 1306 at the 3^rd^ week. Total numbers of spines in experimental group are 1116 at the 1^st^ week, 1610 at the 2^nd^ week, and 1644 at the 3^rd^ week. Error bars indicate mean ± SEM. The significance levels were *p < 0.05, **p < 0.01, and ***p < 0.001. One-way ANOVA Tukey’s multiple comparison tests.

To explore the regulation of the four different types of spines by odor enrichment or odor removal, we calculated the percentages of dendritic spines under the normal raising, odor enrichment, and odor removal conditions. The results showed that long-term odor enrichment induced more mushroom spines (Fig. 3a4) but fewer filopodia and stubby spines (Fig. 3a1,a3). After removing the odor stimulation, the percentage of mushroom spines decreased, while the percentages of thin and stubby spines increased (Fig 3a2,3a3,a4). In the control group, the percentage of filopodia decreased continuously (Fig. 3b1), while thin spines increased slightly at first and remained stable thereafter (Fig. 3b2). The percentages of stubby and mushroom spines remained unchanged (Fig. 3b3,b4).

The stability of the four types of spines was also analyzed throughout the imaging period. Filopodia, the most unstable of the spine types, dropped to 3.72 ± 0.64% in odor enrichment and 4.58 ± 0.78% after odor removal, both markedly lower than in the control group (10.18 ± 2.19%). The stability of stubby spines was found to be 12.12 ± 1.27% in odor enrichment, also significantly lower than in the control group (19.10 ± 2.41%). Meanwhile, compared with the control group (47.17 ± 2.27%), the stability of mushroom spines increased up to 58.27 ± 1.22% in odor enrichment (Fig. 3c).

### Olfactory experiences regulated the spines’ morphological transformation in a short time

Finally, to explore whether the preferences among the 4 types of spines’ morphological transformations were influenced by olfactory experiences, we observed the morphological changes of dendritic spines in the odor stimulation, olfactory deprivation, and control groups at 4-hour intervals and calculated the transformation percentages of the four spine categories. The sensory manipulation was also found to successfully regulate the neural activity of the granule cell in the calcium imaging experiments (Supplementary Fig. S2).

**Figure 4:**
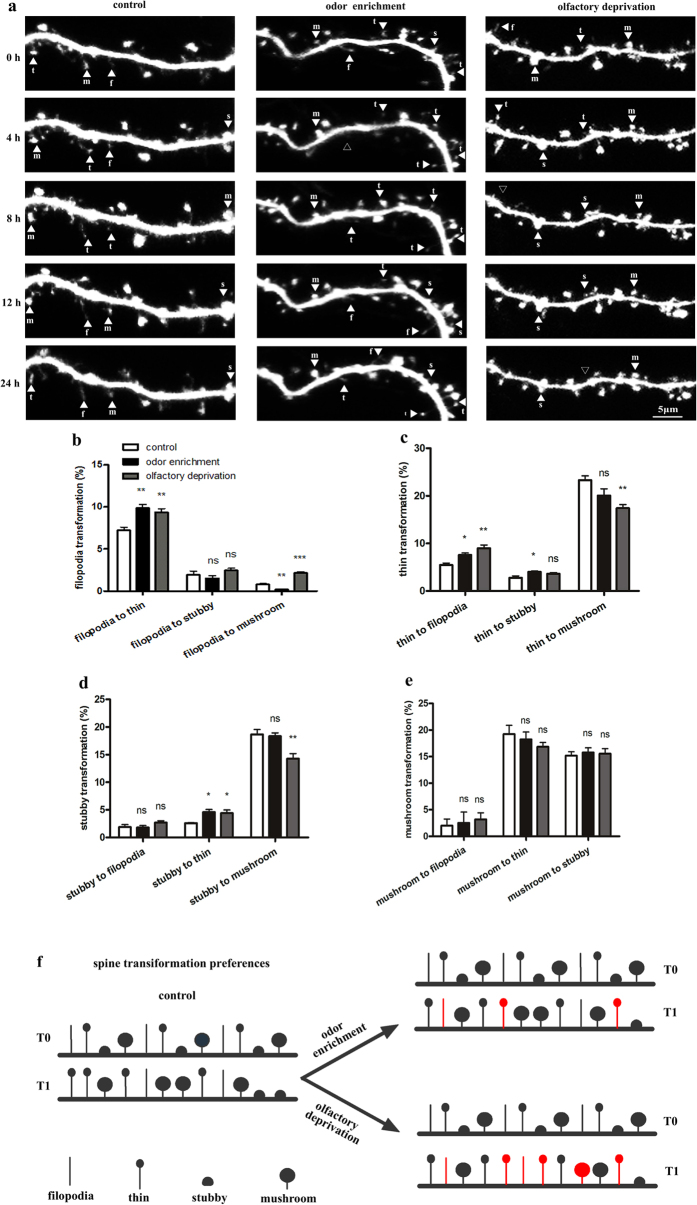
The morphological transformation of spines in different olfactory environments. (**a**) The time-lapse imaging of spines transformed into other types in control, odor enrichment and olfactory deprivation groups. In control group, a thin spine transformed into a mushroom at 4 h, then reverted to a thin at 24 h. A mushroom spine transformed into a thin at 4 h, then transformed into a filopodia at 12 h. In the odor group, a stubby spine transformed into a thin at 4 h, then reverted to a stubby at 12 h. A filopodia spine disappeared at 4 h, then a thin spine appeared at 8 h, transformed into a filopodia at 12 h, and reverted to a thin at 24 h. In the olfactory deprivation group, a filopodia spine transformed into a thin at 4 h, then the thin disappeared at 8 h until 24 h. A thin spine transformed into a stubby at 8 h, then the stubby disappeared at 24 h. Scale bar corresponds to 5 μm. Percentages of filopodia (**b**), thin (**c**), stubby (**d**) and mushroom (**e**) spines that transformed into other types of spines were statistically analyzed. (**f**) Simplified diagram of spine transformation in response to sensory stimulation. For the purpose of illustration, only a sample of spine transformation events is shown and the proportion of spine transformation does not strictly follow the statistical figures. Spines were first imaged at T0 and the spine transformation events happened at T1. The red spines illustrate that the spine transformation preferences were changed. The black spines illustrate the unchanged preferences. “f” indicates filopodia, “t” indicates thin, “s” indicates stubby, and “m” indicates mushroom. The solid arrowhead indicates different spines, and the hollow arrowhead indicates disappeared spines. N = 6 neurons in 6 tadpoles and n = 36 dendrites were used for each group analysis. The total numbers of all transformed spines are 674 in control, 748 in odor enrichment, and 739 in olfactory deprivation. The bars indicate mean ± SEM. Significance was set at *p < 0.05, **p < 0.01, and ***p < 0.001. One-way ANOVA Tukey’s multiple comparison tests.

Spines transformation was indicated by white arrowheads at 0 h, 4 h, 8 h, 12 h, and 24 h in the three conditions (Fig. 4a). In odor stimulation, the percentage of filopodia-to-thin transformation increased to 9.88 ± 0.41%, but filopodia-to-mushroom transformation fell to almost none (0.19 ± 0.07%). Both filopodia-to-thin (9.36 ± 0.43%) and filopodia-to-mushroom (2.18 ± 0.12%) transformation increased after olfactory deprivation (Fig. 4b). Odor enrichment increased the morphological transformation of thin spines, with 7.59 ± 0.41% transformed to filopodia and 4.01 ± 0.17% transformed to stubby spines. Olfactory input deprivation increased the percentage of thin-to-filopodia transformation (8.98 ± 0.67%) but decreased thin-to-mushroom transformation (17.41 ± 0.75%) (Fig. 4c). Odor enrichment increased the percentage of stubby-to-thin transformation (4.60 ± 0.47%). Olfactory deprivation also increased stubby-to-thin transformation (4.41 ± 0.57%), but decreased stubby-to-mushroom transformation (14.28 ± 0.92%) (Fig. 4d). The percentage of mushroom transformation remained unchanged in both odor stimulation and olfactory deprivation (Fig. 4e). All of these data demonstrate that the diverse morphological transformation preferences of the different types of spines were regulated in different ways by olfactory experiences. A diagram also summarized that olfactory experiences facilitated transformation between small spines (thin and filopodia), as well as mutual transformation between the small and large spine types (e.g., stubby to thin, filopodia to mushroom). However, transformation between the large spines (stubby and mushroom) was not influenced by sensory stimulation (Fig. 4f).

Spine addition, elimination, stabilization, and transformation were thoroughly analyzed for a comprehensive picture of the morphological plasticity at 4-hour intervals over 24 h. The data showed that among added spines, the percentage of added stubby spines was reduced in the odor group; filopodia spines were increased but mushroom spines were decreased in the olfactory deprivation group (Supplementary Fig. S3a). On the other hand, there were no differences in spine elimination among the experimental groups and the control group (Supplementary Fig. S3b). As for spine stabilization, stubby spine stability was decreased and mushroom spine stability was increased in odor enrichment; only thin spine stability was increased after olfactory deprivation (Supplementary Fig. S3c). There was no difference in spine transformation between the odor enrichment group and the control group; filopodia spines exhibited much more transformation after olfactory deprivation, while the other types showed no difference (Supplementary Fig. S3d). These results indicate that olfactory experiences had different regulatory effects on the short-term structural plasticity of spines in the granule cell, including addition, elimination, stabilization, and transformation.

## Discussion

The *Xenopus laeves* tadpole has been widely used as an animal model for live imaging due to its good optical transparency in early developmental stages^15^,^20^,^21^. Rodents usually need a thinned-skull or open-skull operation for live imaging, leading to an inflammation response^22^ or increased imaging depth and reduced quality of imaging. By contrast, the olfactory bulb—the transparent and superficial structure in the brain of the tadpole—becomes a unique advantage for the live imaging of individual neurons for noninvasive studies^23^. At the same time, single-cell electroporation labeling allows the dendritic spines of a single neuron to be visualized *in vivo*. Long-term live imaging in the OB in mammals has always been technically challenging, but the imaging methods used in tadpoles make it a much easier process. Compared with previous methods^24^,^25^, *in vivo* time-lapse imaging with subcellular resolution over a period of weeks was accomplished easily in tadpoles in our study, without any surgery or invasive operations.

In order to adjust to novel sensory experiences, odor information processing in the olfactory bulb relies on the synaptic connectivity remolding of interneurons, such as the dendritic spines of granule cells, establishing the framework for connectivity. The morphological plasticity of granule cell dendritic spines induced by olfactory experiences demands more attention. Due to the difficulty in labeling and *in vivo* imaging of olfactory neurons in rodents, few studies were conducted on the spine plasticity of granule cells in the rodent olfactory bulb. Besides, studies on the plasticity of adult-born granule cells in the rodent olfactory bulb^2^,^10^ do not explain how granule cell spines’ plasticity is influenced by long-term odor enrichment or how the morphological transitions among different spines are influenced by short-term olfactory manipulations. Small dendritic spines (filopodia and thin) constantly appear and disappear in a highly dynamic process, identified as the generation of new synapses. Large dendritic spines (stubby and mushroom), associated with memory storage, are relatively stable in the neural network^26^. Our long-term (Figs 1 and 2) and short-term (Fig. 4) *in vivo* imaging provides visual evidence of the regulation of spines’ morphological plasticity by olfactory experiences.

It has commonly been believed that the processes of spine addition, pruning and stabilization are driven by sensory experiences in the rodent cortex^12^,^13^,^27^,^28^. But most of these studies have not distinguished between the different influences that sensory experience exerts on small and large spines. In our *in vivo* imaging, immature small spines turned out to be more dynamic and mature large spines more stable in an odor enrichment environment (Figs 1 and 3c), revealing that the sensory experience-dependent morphological plasticity of spines in the granule cell differed depending on the spine category. Overall, small spines were more dynamic and large spines were more stable, which was consistent with the cortical observations reported in rodents^11^,^29^.

The stable synaptic connection is an important foundation in the mature nervous system^28^,^29^. The increase in the percentage of mushroom spines and decrease in filopodia and stubby spines (Fig. 3a1,3a3,a4) indicated that odor enrichment could promote spines’ maturation. An enriched environment over a period of weeks to months has been reported to promote sensory experience-dependent or motor activity-dependent stability of newly formed spines for lifelong memory storage^9^. Our results show obvious increases in the stability of mushroom spines and decreases in other types of spines (Fig. 3c), indicating that odor enrichment could improve the large spines’ stabilization. Moreover, it is presumable that the stability of the spines mainly comes from the increased percentage of stable large spines induced by odor enrichment, especially mushroom spines (Figs 1e and 3c). However, the partial recovery in the proportion and stability of spines caused by odor removal (Fig. 3a,c) implies that the newly established “memory” mushroom spines induced by odor enrichment are not stable enough. These results suggest that the persistent storage of neural information is based on the maintenance of synaptic connectivity and efficacy, which ought to be based on the structural stability of spines.

The number of dendritic spines and synapse densities can alter under the action of sensory experience. Odor enrichment in tadpoles led to an obvious increase in spine density (Fig. 2d), supporting the idea that the structural dynamics of dendritic spines are influenced by environmental enrichment^30^,^31^,^32^. A point to be noted is that the percentage of mushroom spines decreased, while the percentages of thin and stubby spines increased after odor removal (Fig. 3a2,3a3,a4)—suggesting that the influence exerted by sensory stimulation can be weakened somewhat once the stimulation is removed.

Morphological transitions among different spines can change dynamically in a short time. Among the morphologically altered dendritic protrusions, small spines tend to disappear entirely or transform between filopodia and thin spines, while large spines tend to transform their morphological structures rather than be lost directly^20^. In our study, odor enrichment and olfactory deprivation promoted increased transformation between filopodia and thin spines (Fig. 4b,c), revealing that sensory experiences increase the dynamic property of dendritic spines and result in faster changes in synaptic connections, especially in small spines. The tendency of the highly dynamic filopodia spine to directly disappear or transform into other spines^33^,^34^ explains its gradually decreasing percentage over our 3 weeks of imaging (Fig. 3b1). Additionally, the increasing transformation between the thin and stubby spines (Fig. 4c,d) in odor enrichment suggests that abundant olfactory stimulation facilitates the mutual transformation between the small and large spine types. Meanwhile, the lack of influence exerted by odor enrichment on transformation between stubby and mushroom spines (Fig. 4d,e) implies that the existing large spines are relatively mature, so that moderate and short-term olfactory stimulation is not enough to induce more morphological changes between stubby and mushroom spines. The high number of synaptic vesicles in the presynaptic membrane and high PSD95 in the postsynaptic sides of stubby and mushroom spines^18^,^20^ has also confirmed the presence of mature synapses in these spines. Furthermore, there is no significant difference in the transformation of mushroom spines (Fig. 4e) into the three other types in either odor enrichment or olfactory deprivation, revealing that the mushroom spine is more likely to retain its existing synaptic connections than to be easily affected by olfactory experiences. This is consistent with the low rate of transformation between mushroom spines and other spine categories observed in long-term *in vivo* two-photon imaging over months^19^.

## Conclusions

Taken together, our long-term and short-term *in vivo* imaging have confirmed that olfactory experiences are able to regulate the spine structural plasticity of olfactory bulb granule cells in *Xenopus*, revealing that the morphological plasticity of different types of dendritic spines is the basis of synaptic connectivity and efficacy in the interneurons of the early olfactory system and is vital to olfactory information processing and encoding.

## Methods

### Animals

Adult albino *X. laevis* (Nasco, Fort Atkinson, WI, USA) were induced to ovulate by subcutaneously injecting human chorionic gonadotropin (Sigma Aldrich, St Louis MO, USA). Embryos were obtained by *in vitro* fertilization and maintained in a modified rearing (MR) solution [60 mM NaCl, 0.67 mM KCl, 0.34 mM Ca(NO_3_)_2_, 0.83 mM MgSO_4_, 10 mM HEPES pH 7.4, 40 mg/l gentamycin, pH 7.4~7.6]^35^. All tadpoles were reared under filtered illumination in 12 h dark/light cycles and staged according to staging tables^36^. During imaging and injections, tadpoles were anesthetized with 0.02% ethyl 3-aminobenzoate methanesulfonate (MS222) (Sigma Aldrich, St Louis MO, USA). All the animal manipulations were conducted in strict accordance with the guidelines and regulations set forth by the University of Science and Technology of China Animal Resources Center and University Animal Care and Use Committee; the protocol was approved by the Committee on the Ethics of Animal Experiments of the USTC (Permit Number: USTCACUC1102012).

### *In vivo* single-cell electroporation and single granule cell labeling

The granule cell in the *Xenopus* olfactory bulb was labeled with plasmid DNA by single-cell electroporation *in vivo*^20^,^37^. 0.5–0.8 μg/μl GFP plasmid mixed with 0.05 mM Alexa Fluor^®^ 594 Hydrazide, a red fluorescent dye (A10438, Invitrogen, USA), were co-electroporated into the olfactory granule cell layer. After 2 days, tadpoles with a single spiny neuron expressing GFP throughout their cell bodies, dendrites, and spines were selected for *in vivo* imaging.

### Imaging and data analysis

The morphological changes of the granule cell were followed with time-lapse confocal microscopy in stage 48–51 tadpoles. The anesthetized tadpoles were embedded in low-melting agarose (2%, Sigma) for imaging^20^,^24^. Images were obtained using a water-immersion objective lens (60 × /0.90 w) on a Fluoview FV1000 laser-scanning confocal microscope (Olympus, Japan) equipped with Argon (488 nm excitation). Confocal images of the entire dendritic spines in the neuron were obtained below saturation levels, with minimal gain and contrast enhancements. For the long-term *in vivo* imaging, stacks were collected every day over 5 days and every other day over 3 weeks. For the short-term *in vivo* imaging, stacks were collected at 0 h, 4 h, 8 h, 12 h, and 24 h. Tadpoles were immediately released into MR solution for revival between imaging sessions.

The morphology of spines was divided into four categories—filopodia, thin, stubby, and mushroom—based on the approximate measurements, according to the previously described criteria^38^,^39^,^40^. Filopodia and thin spines were classified as small spines, while stubby and mushroom spines were identified as large spines^29^. Branched spines were not included in the statistics, given the limited number of cases.

The entire lengths of dendrites were used for quantitative analysis. All image analysis was based on the raw data of confocal imaging. Digital three-dimensional reconstructions of confocal stacks were obtained from individual optical sections using Imaris 7.0.1 (Bitplane, Switzerland). Thin optical sections (1.0 μm) through the entire extent of the dendrite were collected to gain high image quality. The values of green channel pixel intensity were between 50 and 255. The three-dimensional projection could be freely rotated and magnified using the Surfaces module to view the spines on the Z-axis or behind the overlap region. The definitions and classifications of spine morphology were applied through the Imaris Filament module. The AutoPath mode of the Filament module was used to trace the dendritic arbors of a neuron. The Manual option was used to trace dendritic spines based on the dendritic framework, with the four different kinds of spines assigned four different colors.

Several index parameters were measured and calculated to obtain detailed information on the 4 types of dendritic spines at each time interval: spine density (the number of spines per unit arbor length), spine addition (the proportion of added spines compared to previously observed ones), spine elimination (the proportion of eliminated spines compared to previously observed ones), spine dynamics (the mean value of spines addition and spines elimination), spine stability (the proportion of stable spines compared to previously observed ones). The number following the ± sign was a standard error (SEM). Statistical analyses were reported as mean ± SEM using two-sample unpaired t-tests and one-way ANOVA Tukey’s multiple comparison tests (GraphPad Prism 5.0, USA), with a significance of p < 0.05 indicated by an asterisk, p < 0.01 indicated by two asterisks, and p < 0.001 indicated by three asterisks.

### Odor stimulus application

As odorants, amino acids (Supplementary Table S1) were dissolved in the MR solution to create 200 μM work solution. All of the amino acids were used in one mixture for odor stimulation^41^,^42^,^43^,^44^. For the long-term morphological imaging, tadpoles were continuously exposed for several days, and a static-renewal procedure was applied, with complete odor solution exchange every 24 h.

### Olfactory deprivation

After tadpoles were anesthetized, tiny fissures on the skin over the olfactory nerves were made with a silver needle under a binocular stereo microscope (Olympus, Japan). Then, the olfactory nerves were cut off completely using Vannas scissors (66VT, China) to block sensory input to the olfactory bulb. After surgery, specimens were reared in MR solution for recovery.

## Additional Information

**How to cite this article**: Zhang, L. *et al*. Olfactory experiences dynamically regulate plasticity of dendritic spines in granule cells of *Xenopus* tadpoles *in vivo*. *Sci. Rep*. **6**, 35009; doi: 10.1038/srep35009 (2016).

## Supplementary Material

Supplementary Information
