# Supplementary material for: Olfactory experiences dynamically regulate plasticity of dendritic spines in granule cells of Xenopus tadpoles in vivo
Source: Sci Rep. 2016 Oct 7;6:35009. doi: 10.1038/srep35009 (PMC5054522; doi:10.1038/srep35009)
Supplement: Supplementary Information [file srep35009-s1.doc]

**Olfactory experiences dynamically regulate plasticity of dendritic spines in granule cells of *Xenopus* tadpoles *in vivo***

**Li Zhang, Yubin Huang, Bing Hu***

Chinese Academy of Sciences Key Laboratory of Brain Function and Disease, and School of Life Sciences,

University of Science and Technology of China, Hefei, Anhui Province, P. R. China

* Corresponding author

E-mail: [bhu@ustc.edu.cn](mailto:bhu@ustc.edu.cn)

**Supplementary results of calcium imaging.**

We used GCaMP6f as the calcium indicator to test whether the instantaneous activities of dendritic spines in the granule cell were regulated by different olfactory experiences, like odor stimulation or olfactory deprivation. We tracked the vehicle-stimulated, odorant-stimulated, and olfactory deprivation-induced calcium signal dynamics of the four types of dendritic spines through in vivo calcium imaging in awake tadpoles. The results indicated that odor enrichment and olfactory deprivation facilitated and suppressed, respectively, the calcium responses of dendritic spines in the granule cell (Supplementary Fig. S2). Thus, we have demonstrated that sensory manipulation can successfully regulate the neural activity of the granule cell.

**Supplementary methods of calcium imaging and data analysis.**

GCaMP6f[^1^](#_ENREF_1) was used to label a single granule cell for in vivo calcium imaging. In order to improve the expression efficiency of GCaMP6f, UAS-ks-GCaMP6f and CMV: pCS2-Gal4 plasmids mixed with Alexa Fluor® 594 Hydrazide were co-electroporated. Imaging of dendrite spines’ calcium signal acquisition was performed under a water-immersion objective lens (60×/0.90 w) on the confocal microscope. Two-dimensional confocal imaging was acquired at 3 Hz. The partial enlarged image was chosen from the intact reference image with 1600×1600 pixel frames. In order to keep the tadpoles awake, we used pancuronium bromide—an aminosteroidal competitive neuromuscular blocker—to block the sensory-motor responses during the live imaging procedure. To observe odor-evoked calcium responses in the neural spines, we applied odorants to the olfactory epithelium while taking sequences of images of the spines. To investigate the calcium signal dynamics after olfactory deprivation, we collected time series of the calcium images after severing the olfactory nerves. For the short-term calcium imaging, the odor solution was applied directly to the olfactory epithelium for 5 minutes; in the vehicle control group, MR solution was applied to the olfactory epithelium for 5 minutes.

The time series image stacks of spines’ calcium signals were exported as TIFF files into ImageJ plugins for analysis. The individual regions of interest (ROIs) were manually selected from movies displaying fluorescence changes at each time point. A StackReg plugin (available from public website http://rsbweb.nih.gov/ij/index.html) was used for the recursive alignment of a stack of images to correct image position offset in movies. Calcium signal amplitudes were given as average ΔF/F values (ΔF/F= (F_t_−F_0_)/F_0_), with the average fluorescence intensity in the baseline period (10 frames) defined as F_0_ and the average fluorescence intensity within the ROI at time t defined as F_t_. Average fluorescence intensity was examined during the 300 s acquired in calcium imaging.

**References:**

1. Chen, T.W. et al. Ultrasensitive fluorescent proteins for imaging neuronal activity. *Nature* **499**, 295-+ (2013).

2. Ramlochansingh, C., Branoner, F., Chagnaud, B.P. & Straka, H. Efficacy of Tricaine Methanesulfonate (MS-222) as an Anesthetic Agent for Blocking Sensory-Motor Responses in Xenopus laevis Tadpoles. *Plos One* **9** (2014).

3. Bartlett, H.L., Scholz, T.D., Lamb, F.S. & Weeks, D.L. Characterization of embryonic cardiac pacemaker and atrioventricular conduction physiology in Xenopus laevis using noninvasive imaging. *American Journal of Physiology-Heart and Circulatory Physiology* **286**, H2035-H2041 (2004).

**Supplementary Figures.**


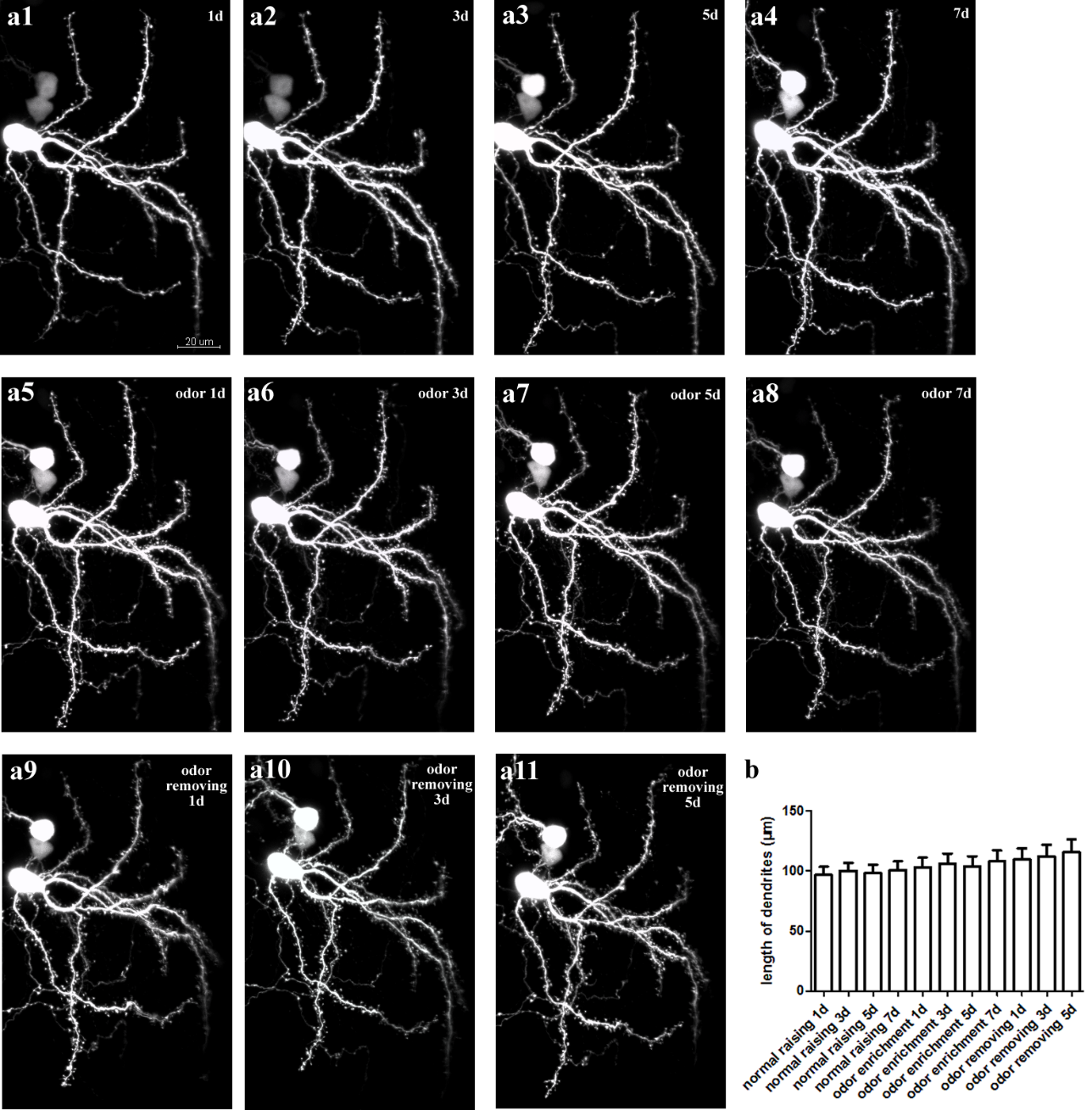


**Supplementary Fig. S1 Long-term imaging of granule cell dendrites *in vivo*.**

(**a**) Time-lapse imaging of dendrites in experiment group (normally raising for 1 week, odor treating for 1 week, and odor removing for 1 week) showed the branching number and pattern of dendrites were not changed by olfactory experience. (**b**) Quantitative analysis of dendrite length showed no significant difference during the three weeks of imaging. N = 6 neurons in 6 tadpoles and n = 21 dendrites were used for the 2-day interval observation.

**
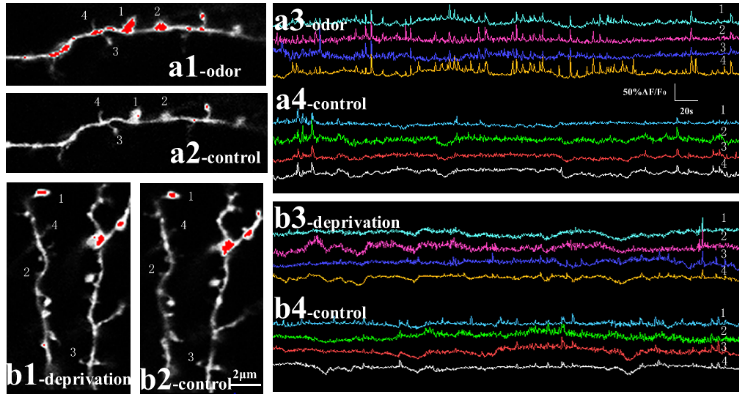
**

**Supplementary Fig. S2 Calcium transients of the spines in the granule cell regulated by odor stimulation and olfactory input deprivation in awake tadpoles.**

(**a**, **b**) In vivo calcium imaging in odor stimulation (**a1**) versus control (**a2**) and olfactory deprivation (**b1**) versus control (**b2**) were traced, and calcium transient dynamics were separately exhibited in **a3**, **a4**, **b3**, and **b4**. Scale bar corresponds to 2 μm. ΔF/F refers to measured fluorescence normalized to background fluorescence.


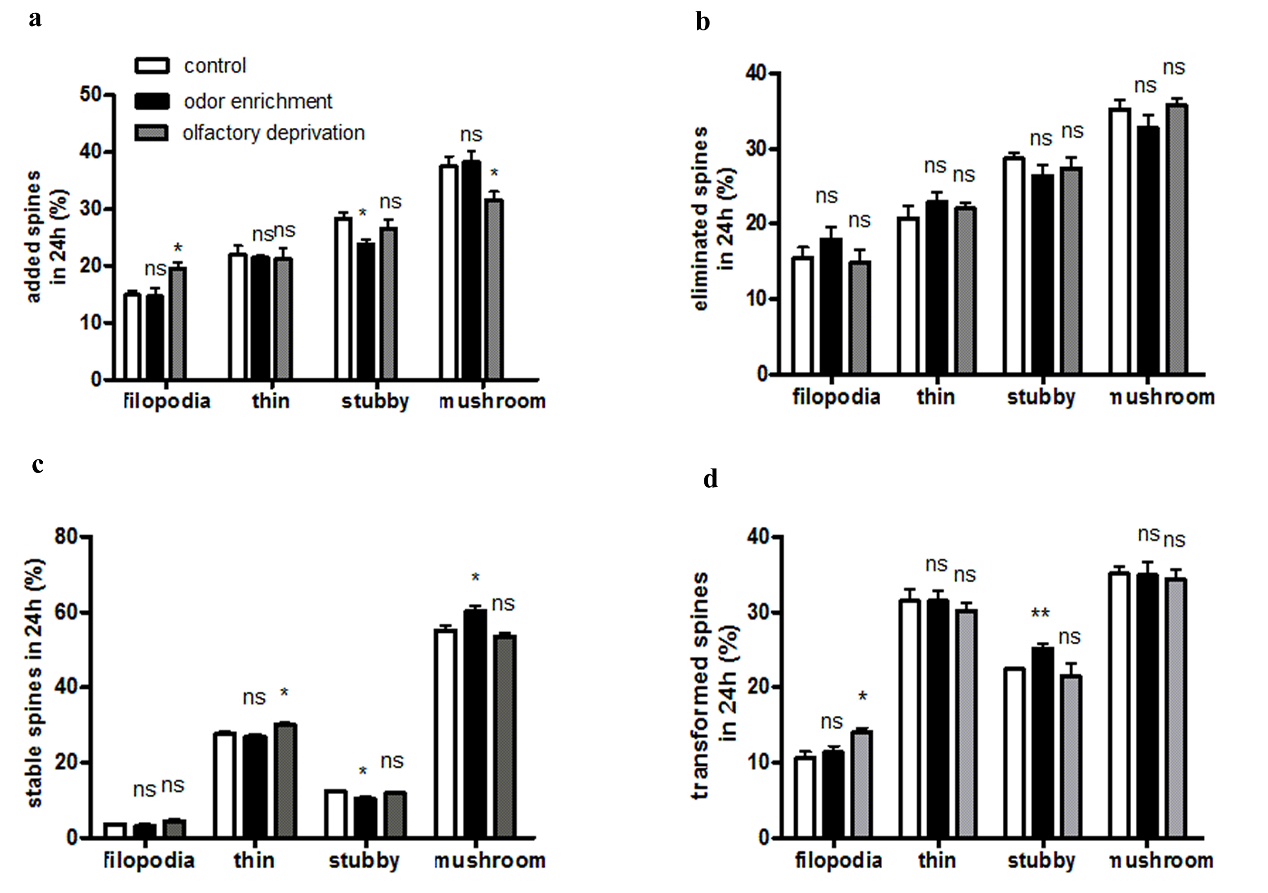


**Supplementary Fig. S3 Spine plasticity influenced by olfactory experiences from short-term imaging.**

Quantitative analysis of added (**a**), eliminated (**b**), stable (**c**), and transformation (**d**) of four types of spines in 24 h showed spines were differently regulated by olfactory experiences. N = 6 neurons in 6 tadpoles and n = 36 dendrites were used for each group analysis. The total numbers of spines are 2558 in control, 2731 in odor enrichment, and 2606 in olfactory deprivation. The bars indicate mean ± SEM. Significance was set at *p < 0.05, **p < 0.01, and ***p < 0.001. One-way ANOVA Tukey’s multiple comparison tests.
